# The Impact of Visual Art and High Affective Arousal on Heuristic Decision-Making in Consumers

**DOI:** 10.3389/fpsyg.2020.565829

**Published:** 2020-11-26

**Authors:** Yaeri Kim, Kiwan Park, Yaeeun Kim, Wooyun Yang, Donguk Han, Wuon-Shik Kim

**Affiliations:** ^1^Department of Digital Marketing, School of Management, Sejong Cyber University, Seoul, South Korea; ^2^Department of Marketing, Business School, Sejong University, Seoul, South Korea; ^3^Department of Marketing, Business School, Seoul National University, Seoul, South Korea; ^4^Department of Marketing, Orfalea College of Business, California Polytechnic State University, San Luis Obispo, CA, United States; ^5^Department of Marketing, Hong Kong University of Science and Technology, Kowloon, Hong Kong; ^6^Center for Medical Convergence Metrology, Korea Research Institute of Standards and Science, Daejeon, South Korea; ^7^Future Medicine Division, Korea Institute of Oriental Medicine, Daejeon, South Korea

**Keywords:** visual arts, affective arousal, consumer, decision-making, EEG, Stroop task

## Abstract

In marketing, the use of visual-art-based designs on products or packaging crucially impacts consumers’ decision-making when purchasing. While visual art in product packaging should be designed to induce consumer’s favorable evaluations, it should not evoke excessive affective arousal, because this may lead to the depletion of consumer’s cognitive resources. Thus, consumers may use heuristic decision-making and commit an inadvertent mistake while purchasing. Most existing studies on visual arts in marketing have focused on preference (i.e., affective valence) using subjective evaluations. To address this, we applied a neuroscientific measure, electroencephalogram (EEG) to increase experimental validity. Two successive tasks were designed to examine the effects of affective arousal and affective valence, evoked by visual artwork, on the consecutive cognitive decision-making. In task 1, to evaluate the effect of visual art, EEG of two independent groups of people was measured when they viewed abstract artwork. The abstract art of neoplasticism (AbNP) group (*n* = 20) was showing Mondrian’s artwork, while the abstract art of expressionism (AbEX) group (*n* = 18) viewed Kandinsky’s artwork. The neoplasticism movement strove to eliminate emotion in art and expressionism to express the feelings of the artist. Building on Gallese’s embodied simulation theory, AbNP and AbEX artworks were expected to induce lower and higher affect, respectively. In task 2, we investigated how the induced affect differentially influenced a succeeding cognitive Stroop task. We anticipated that the AbEX group would deplete more cognitive resources than AbNP group, based on capacity limitation theory. Significantly stronger affect was induced in the AbEX group in task 1 than in the AbNP group, especially in affective arousal. In task 2, the AbEX group showed a faster reaction time and higher error rate in the Stroop task. According to our hypotheses, the higher affective arousal state of the AbEX group might deplete more cognitive resources during task 1 and result in poorer performance in task 2 because affect impacted their cognitive resources. This is the first study using neuroscientific measures to prove that high affective arousal induced by visual arts on packaging may induce heuristic decision-making in consumers, thereby advancing our understanding of neuromarketing.

## Introduction

The application of visual art-based designs to products or their packaging greatly impacts the purchase-related decision-making of consumers. The phenomenon of “art infusion” (Hagtvedt and Patrick, 2008, 2011), whereby perceptions of visual art spill over onto an object with which the art is associated, has a favorable effect on the consumer product evaluation. For example, art image (vs. non-art image) printed on the packaging caused a spillover of luxury perceptions and resulted in favorable evaluations of the product by the consumers (Hagtvedt and Patrick, 2008).

When we appreciate a work of figurative art, besides deriving meaning, we may also feel the affect expressed by the artist through the artwork (Uusitalo et al., 2012). This affective influence is grounded in embodied simulation theory, which posits that the emotions and sensations of the artist can be simulated within the visceromotor and somatosensory systems of the viewer (Gallese and Sinigaglia, 2011). Interestingly, an artwork may elicit affect among viewers even in the absence of clear figurative content, as in the case of abstract art (Freedberg and Gallese, 2007). There are two opposing art movements in abstract art: neoplasticism and expressionism. Although subsumed under the same label of abstract art, the neoplasticism movement, led by Mondrian, tried to eliminate emotion from art, whereas the expressionism movement, led by Kandinsky, insisted that art should express the feelings of the artist (Strickland and Boswell, 1992; Melcher and Bacci, 2013; van Paasschen et al., 2015).

To evaluate the affect induced in viewer, a two-dimensional model of affect including independent measures of affective valence and affective arousal has widely been adopted by psychologists (Russell and Barrett, 1999; Schmidtke and Heller, 2004). According to the lateralization of emotional processes in the frontal lobes (Ahern and Schwartz, 1985; Davidson and Irwin, 1999; Grimm et al., 2006; Cheung et al., 2019), positive valence activates the left dorsolateral prefrontal cortex (DLPFC), while negative valence activates the right DLPFC. Affective arousal activates the right parietotemporal (PT) region (Heller et al., 1997). This two-dimensional affect can be measured using EEG based on a negative correlation between regional cortical activation and EEG alpha power (Coan and Allen, 2004; Oakes et al., 2004). Due to the limited spatial resolution of EEG, researchers have started using functional magnetic resonance imaging (fMRI) to precisely localize the cortical sources of valence-related activation. The results of their studies are consistent with the lateralization of emotional processes in the prefrontal cortex, i.e., the left DLPFC is activated for positive valence and the right ventrolateral prefrontal cortex (VLPFC) is activated for negative valence. Additionally, within the prefrontal cortical region, the dorsomedial prefrontal cortex (DMPFC) is activated during affective arousal (Dolcos et al., 2004; Grimm et al., 2006). To evaluate the affect induced by a visual art stimulus, positive frontal alpha asymmetry using EEG (i.e., positive valence responses) was observed, regardless of whether it was perceived as beautiful. However, when evaluating commercial stimuli, this positive asymmetry was elicited only when the stimulus was considered beautiful (Cheung et al., 2019). This can be explained by self-rewarding characteristics of aesthetic experiences that may impact the beholder’s affective state (Mastandrea et al., 2019). The frontal alpha asymmetry is also associated with several brain functions such as personality (Schmidtke and Heller, 2004) and working memory (Bacher et al., 2017).

The Heuristic-Systematic dual-processing model (Chaiken, 1980) states that consumers tend to process information systematically when there is motivation and ability to process the information. Such processing includes an analytic orientation in which consumers carefully examine all task-relevant information. However, if there is a low motivation or the capacity to process information is constrained or the available time is less, irrespective of the motivation, the heuristic processing (characterized by effort-reduction and limited consideration of information) is executed. That is, the available time is similar to the cognitive capacity or the ability to process information (Suri and Monroe, 2003). In the study of the influence of affect on decision-making, a considerable number of studies have shown that a positive mood tends to trigger heuristic processing when making judgments, while a negative mood leads to systematic processing (Bless et al., 1996; Elsbach and Barr, 1999; Park and Banaji, 2000; Mitchell and Phillips, 2007). Furthermore, according to capacity limitation theory (CLT), which posits cognition and affect share the same cognitive resources (Mackie and Worth, 1989; Seibert and Ellis, 1991; Mitchell and Phillips, 2007), heuristic processing is appropriate when fast decisions are required, i.e., under time constraint, and when there is a shortage of processing capacity. Thus, it tends to result in poorer decision-making performance (Suri and Monroe, 2003; Shah and Oppenheimer, 2008). As affect consists of affective valence and affective arousal, visual artwork inducing either high affective valence or high affective arousal may deplete the viewer’s cognitive resources while processing, thereby resulting in poorer performance in the subsequent cognitive task. To impact consumer’s purchase-related decision-making, visual art should be designed to facilitate systematic decision-making as well as evoke aesthetic preference. Heuristic decision-making may lead to errors in purchasing resulting in regret. Most existing studies have focused on the effect of art associated with a product from a viewpoint of aesthetic preference or favorable evaluation (Hagtvedt and Patrick, 2008, 2011). Moreover, the literature exploring the influence of the affect induced by visual stimuli on the cognitive bases of decision-making has primarily focused on mood or affective valence while neglecting affective arousal (Hagtvedt and Patrick, 2008; Mastandrea et al., 2019). Therefore, this study aims to examine whether opposing styles of visual art (expressing vs. suppressing) on product packaging impact the consumers’ affect differently. If this proves to be the case, we intended to investigate how the differential changes in consumers’ affect induced by the two styles of visual art, influence the way information is processed in purchase decision-making. We chose abstract art instead of figurative art, because the latter may drive some meaning which will impact the viewers’ affect. We measured the affect induced by abstract art by using 128-channel EEG as well as subjective evaluation. Color word Stroop task was used as the cognitive task, since it has been widely used to evaluate the effects produced after affect has been induced (Brand et al., 1997).

We hypothesized that the affect induced in consumers who viewed abstract art of expressionism (AbEX) is greater than who viewed abstract art of neoplasticism (AbNP) based on embodied simulation theory, because the expressionism artists poured affect much more to their artworks as opposed to neoplasticism artists. Thus, the consumers who view AbEX (vs. AbNP) can imagine the artist’s emotional and gestural movement more strongly. This greater affect induced in consumers who viewed AbEX (vs. AbNP) could cause a depletion of cognitive resources by the CLT and compel them to adopt heuristic information processing, grounding on Heuristic-Systematic dual-processing model, thereby resulting in poorer performance in the subsequent cognitive task.

## Materials and Methods

### Participants

To verify our hypotheses, we recruited 50 healthy, right-handed participants. Even though there are sex differences in behavior and cognition based on sexual dimorphism (e.g., Kandel, 1999, 2012), only male participants with short hair were recruited so as to maintain homogeneity and since short hair takes lesser time to adjust each of the 128 electrodes perpendicular to the scalp in the 128-channel HydroCel Geodesic Sensor Net (see Section “EEG recording”). All participants had normal or corrected-to-normal vision and were undergraduate students from the South Korea. The participants were not artists since the cortical reaction of artists is different from the non-artists when exposed to artworks (Bhattacharya and Petsche, 2005; Seeley and Kozbelt, 2008; Kottlow et al., 2011).

They were randomly divided into two experimental groups: one group was showing Mondrian’s abstract artwork (AbNP group), and the other, Kandinsky’s abstract artwork (AbEX group) (see Section “Viewing the abstract artwork (task 1)”). We excluded data from 12 of the participants due to excessive EEG artifact, potential sleepiness, malfunctions of the EEG system, and extreme outliers as estimated by box plots. After these exclusions, data from 20 AbNP group participants (age: *M* = 24.45, *SD* = 2.24) and 18 AbEX group participants (age: *M* = 23.72, *SD* = 2.45) were used in further analysis. No significant difference was found in age (*F* < 1) between the two groups. Informed consent was obtained from all participants, and they were compensated (W== 30,000; approximately $30) for completing the study. This study was approved by the Institutional Review Board at the Korea Research Institute of Standards and Science (KRISS-I-15-1) and was carried out in accordance with the provisions of the World Medical Association Declaration of Helsinki.

### Procedure and Experimental Setup

#### Procedure

The experiment consisted of two consecutive tasks, i.e., viewing the abstract artwork (task 1) and performing the Stroop task (task 2). It was conducted in a dimly lit, acoustically and electromagnetically shielded room. Participants were made to sit on comfortable armchairs in front of a 23-in. computer monitor placed at a distance of approximately 67 cm from their eyes. Prior to the experiment, the participants were familiarized with the Stroop task. Next, a 128-channel Hydrocel Geodesic Sensor Net (HCGSN) (see Section “EEG recording”) in one of three sizes (small: 54–66 cm, medium: 56–58 cm, large: 58–61 cm) depending on each participant’s head circumference, was applied and the impedance was checked. In task 1, we measured the differences in affect induced by contrasting artworks, i.e., AbNP vs. AbEX, using EEG. In task 2, we measured the participants’ reaction time (*RT*) and error rate (*ErR*) while they were performing the Stroop task but did not measure EEG because there was severe motion artifact. Additionally, since we used the 128-channel dense HCGSN, which uses a saline solution to reduce the impedance between the electrode and the scalp, the electrical noise between these substances increased as the saline dried out (see Section “EEG recording”). Between tasks 1 and 2, it was impractical to check the impedance or refill the saline solution since the difference of affect induced by the two opposing styles of abstract art would then be diminished. Therefore, task 2 was designed to follow task 1 immediately without checking the impedance between tasks 1 and 2, and thus, without measuring EEG.

**FIGURE 1:**
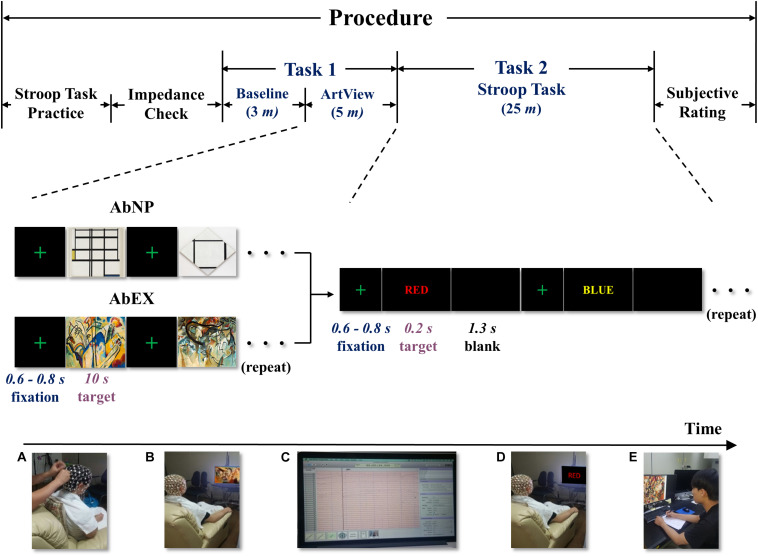
The experimental procedure including tasks 1 and 2: bottom left to right, **(A)** applying sensor net and checking impedance, **(B)** viewing abstract artwork, **(C)** acquiring EEG signals, **(D)** performing the Stroop task, and **(E)** subjective ratings for abstract artwork. ArtView, artwork-viewing session; AbNP, artwork of Mondrian in the style of neoplasticism; AbEX, artwork of Kandinsky in the style of expressionism.

Visual stimuli, both the abstract art and the Stroop task, were programmed and presented via E-Prime^®^ 2.0 (Psychology Software Tools Inc., Pittsburgh, Pennsylvania), a stimulus software compatible with the EEG data acquisition software Net Station 5.2 (EGI, Eugene, OR, United States). To assess the subjective evaluation of visual stimuli more objectively, two manipulation check groups were recruited (Perdue and Summers, 1986), consisting of male participants for AbNP group (*n* = 33; age: *M* = 27.91, *SD* = 4.11) and AbEX group (*n* = 33; age: *M* = 28.45, *SD* = 4.22). While observing each piece of abstract art, the participants responded to a series of questions on a 9-point Likert scale ranging from strongly disagree to strongly agree, including questions around affective valence (“This artwork makes me feel positive”), affective arousal (“This artwork makes me feel aroused”), dynamicity (“This artwork makes me feel dynamic”), complexity (“This artwork is highly complex”), and emotional infusion (“I can feel great amount of emotional properties”). The average duration of the experiment per participant was 1 h (see Figure 1).

#### Experimental Setup

##### Viewing the abstract artwork (task 1)

**FIGURE 2:**
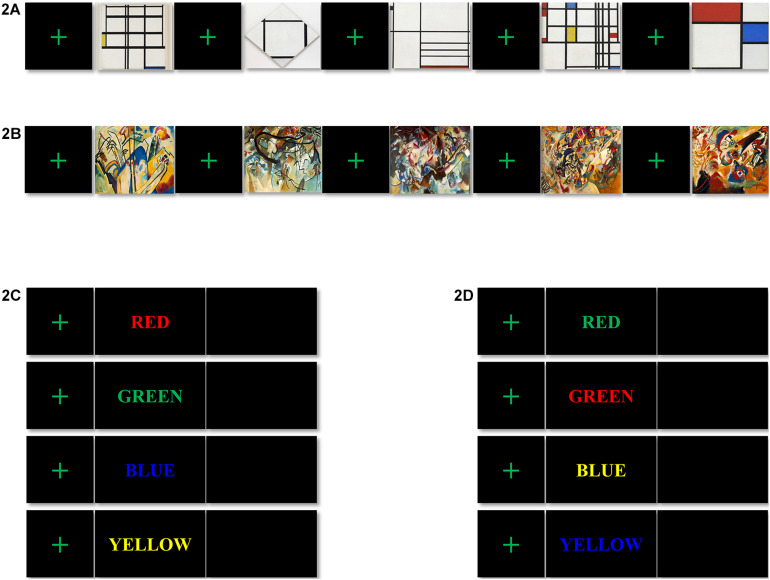
Stimuli for tasks 1 and 2: the visual stimuli for task 1, **(A)** artwork of Mondrian in the style of neoplasticism (AbNP); from left to right, 1937 *Composition I in Yellow, Blue, and White*, 1926 *Tableau I, Lozenge with Four Lines and Gray*, 1936 *Composition in White, Black, and Red*, 1937–42 *Composition in Red, Blue, and Yellow*, and 1929 *Composition No. II, with Red and Blue*, and **(B)** artwork of Kandinsky in the style of expressionism (AbEX); from left to right, 1911 *Composition IV*, 1911 *Composition V*, 1913 *Composition VI*, 1913 *Composition VII*, and 1913 *Fragment 2 for Composition VII*. The color-word stimuli for task 2: **(C)** congruent color-word stimuli; **(D)** incongruent color-word stimuli.

Task 1 lasted for approximately 8 min, including a baseline session and an ArtView session. After completing the 3-min baseline session, the participants moved on to the 5-min ArtView session. For the purpose of experimentally inducing affect, we used abstract artwork from the Website of the Museum of Modern Art (MOMA^1^). Even though the international affective picture system (IAPS) is a highly replicated data set associated with known stimuli valence-rated set of images, it was not used in the current study since it consists of pictures and not artworks. In this study, we want to examine how visual art (not picture), used in product packaging design influences the viewer’s decision-making. We chose abstract art instead of figurative art, because the latter may drive some meaning that can modulate the viewer’s affect. Specifically, artworks of two leading abstract artists, Mondrian and Kandinsky, were selected because the former exemplifies AbNP and the latter AbEX (Brandabur, 1973; Strickland and Boswell, 1992; Simon, 2013). Before starting task 1, the saline solution was reduced to avoid excess saline solution from dripping down the participants’ foreheads causing annoyance, and thus influencing sensible human affect. Thus, to prevent the drying out of the saline solution, we designed task 1 to be 10 min in duration (see Sections “Procedure” and “EEG recording”). We decided to select five abstract artworks for each group—AbNP and AbEX, which were *1937 Composition I in Yellow, Blue, and White*, *1926 Tableau I, Lozenge with Four Lines and Gray*, *1936 Composition in White, Black, and Red*, *1937–42 Composition in Red, Blue, and Yellow*, and *1929 Composition No. II, with Red and Blue* for AbNP (see Figure 2A) and *1911 Composition IV*, *1911 Composition V*, *1913 Composition VI*, *1913 Composition VII*, and *1913 Fragment 2 for Composition VII* for AbEX (see Figure 2B). These five consecutive sets, in which a fixation screen was followed by an image, were presented four times to each participant, i.e., 20 stimuli per participant (see Figure 1). This study focused on how the opposing styles of artwork influence the cognitive task, i.e., cognitive decision-making. Participants were asked to rest their forearms on the armrest of the chair. In addition, during the baseline session, the participants were asked to relax both their mind and body and avoid ideas and thoughts as much as possible. During the ArtView session, participants were expected to first fixate on a cross at the center of the monitor, and then observe a series of abstract paintings belonging to either AbNP or AbEX, depending on their assigned group. Throughout the observation process, participants were asked to focus on the images as much as possible. Each block in the experimental trials consisted of five consecutive sets. Each image was shown for 10 s, while the duration of the fixation cross varied from 0.6 to 0.8 s to reduce the predictability of the task and maintain the participants’ attention. Completing one block took approximately 53.5 s (i.e., ≃(0.7 + 10.0 s) × 5). Participants completed the same block four times, separated by 5-s intervals. Thus, the ArtView session took approximately 5 min total (i.e., ≃(53.5 + 5 s) × *4-5* s) (see Figures 1, 2).

##### Performing the stroop task (task 2)

For task 2, a color-word Stroop task was employed because it has been considered the golden standard for attentional measure (MacLeod, 1992). Furthermore, the Stroop task has widely been used to evaluate the effects produced after affect has been induced (Brand et al., 1997). Task 2 lasted approximately 25 min (see Figure 1). Eight different color words were used as stimuli and were written in four colors (red, green, blue, and yellow) on a black background. Four of the color words were used for congruent stimuli (e.g., the word “RED” written in red), and the other four for incongruent stimuli (e.g., the word “RED” written in green). Participants were asked to look at the color words and press the button marked “1” for congruent or “2” for incongruent as accurately and as quickly as possible, i.e., under time constraint. Each stimulus in task 2 was again preceded by a fixation cross at the center of a black screen. Each small block consisted of eight consecutive trials in which a fixation cross was followed by a congruent or incongruent color word, presented in a random order, followed by a blank screen. While the duration of the fixation cross varied in the same way as was done for task 1 (i.e., it was presented for 0.6–0.8 s), the duration of each color word and the blank screen was set for 0.2 and 1.3 s, respectively. Therefore, one small block took approximately 17.6 s (i.e., ≃(0.7 s + 0.2 + 1.3 s) × 8). A large block of 20 small blocks was repeated four times with 15-s intervals. It took almost 25 min (i.e., ≃(17.6 × 20 + 15 s) × *4-15* s) to complete task 2. Each participant saw 640 color words ((8 × 20) × 4 = 640), where the congruent and incongruent words were equally distributed (320 times each) and presented in a random order (see Figures 1, 2).

### Data Recording and Analyses

#### EEG Recording and Analyses

##### EEG recording

During task 1, EEG data was recorded using a 128-channel Hydrocel Geodesic Sensor Net (HydroCel GSN (HCGSN) 130, Electrical Geodesics, Inc. [EGI], Eugene, OR, United States) and amplified by Geodesic EEG System 400 (Net Amps 400, EGI, Eugene, OR, United States). Before applying the 128-channel dense HCGSN to participant’s head, the HCGSN was soaked in electrolyte for at least 5 min to improve conductance and ensure adequate wetting of the sponges attached to electrodes. Generally, with enough electrolytes in the sponge, the HCGSN can be used for a 2-h recording. However, trickling of excessive electrolyte on participants’ forehead will have a serious influence on sensible human affect. Therefore, the electrolyte in the sponge was reduced in this study, thus increasing the impedance between the electrode and scalp dramatically as the solution dries out. To reduce the impedance, the 128 electrodes were adjusted perpendicular to the participant’s scalp. To save time, male participants with short hair were recruited as opposed to female participants with long hair. All electrodes were referenced to Cz and the impedances were checked to ensure that they were less than 50 kΩ. The EEG data were digitized using a 24-bit A/D converter at a 1-kHz sampling rate with an online band-pass filter set at 0–400 Hz, and analyzed using EGI acquisition software (Net Station version 5.2, EGI, Eugene, OR, United States) on an Apple Mac Pro computer running on OS 10.5.5. To minimize EEG artifact associated with eye movements such as blinking, we asked the participants to keep their eyes open, stay alert, and not move their body unless necessary during both the baseline and ArtView sessions.

##### EEG analysis

The data were filtered offline by a 0.5–50-Hz band-pass filter using Net Station S/W and down-sampled to 250 Hz. Next, 30 stimulus-locked segments (each 10-s long), consisting of 10 from the baseline session and 20 from the ArtView session, were extracted per participant, since sample sizes equal to or greater than 30 have normal distribution as per the central limit theorem (Moen and Powell, 2005). If we increase the length of the EEG segment, i.e., the bin size, the number of specific waves in the segment will increase along with the probability of including noise. We want a large enough number to capture the specific wave, while excluding noise. To decide the optimal bin size, we made a trade-off between the sample size balancing signal and noise. In this study, we were interested in the theta, alpha, and beta bands. Therefore, the frequency of interest of EEG ranges from 4 to 32 Hz. For the lowest frequency in this (4 Hz), almost 40 cycles of the wave are included in a 10-s segment of EEG. For frequencies above 4 Hz, the sample size will be larger. Therefore, we set the bin size as 10 s. Below 10 s, the reliability of theta band power measurements would be severely degraded and above 10 s, the reliability of power measurements of the three frequency bands would reduce due to noise. In task 1, the time to show one abstract art was also 10 s (see Figure 1). To reject artifacts resulting from eye blinks or movements from the EEG, a validated method based on an independent component analysis algorithm from the EEGLAB toolbox (Delorme and Makeig, 2004; Gabard-Durnam et al., 2018) was used. We analyzed the artifact-free EEG traces by applying a fast Fourier transform (FFT) with a Hamming window using MATLAB R2014a (MathWorks, Natick, MA, United States). When we inspected the frequency characteristics of a specific EEG segment, we transformed the EEG from the time domain to the frequency domain with the FFT. Since the EEG returns non-stationary signals, the frequency characteristics will vary during the 10-s interval. If we apply the FFT to the full length of the EEG segment, we will not know how the frequency characteristics change over the 10-s interval. Therefore, we apply the FFT over short intervals. The width of the window is the length of the short time interval to which the FFT is applied. In this study, we assumed it as 1 s. There are different windows used to reduce spectral leakage when performing a FFT on time data to convert it into the frequency domain. The Hamming window is a taper formed with a raised cosine with non-zero endpoints, optimized to minimize the nearest side lobe. The purpose of overlapping is to reduce the effects of windowing. Most windowing functions (e.g., Blackman, Hamming) are taper-shaped, which means that they drop to 0 (or close to 0) near the frame edges. This of course affects the FFT results and can lead to the loss of some important information (e.g., transients). Thus, overlapping is used to reduce the negative effect of windowing. In this study, we chose a 50% overlapping, i.e., a 0.5-s overlapping for a 1-s window. Finally, 19 windows were left with 1-s width in a 10-s segment, because the width of the 20th window is only 0.5 s.

To inspect the regional cortical activity around each electrode on the scalp, we calculated the mean powers in the theta (*P(θ)*, 4–8 Hz), alpha (*P(α)*, 8–14 Hz), and beta bands (*P(β)*, 14–32 Hz) for each window and averaged these over the 19 windows. We averaged the mean powers over the artifact-free 10-s segments for each of the 128 electrodes. We defined the average mean power at each electrode *n* in the theta, alpha, and beta bands as, *P*_*n*_(θ), *P*_*n*_(α), and *P*_*n*_(β), respectively. Next, to evaluate regional cortical activity around each scalp electrode, we defined the mean power ratio at electrode *n* as *P*_*n*_(*r*) (see Eq. (1) below for reference). In Eq. (1), *P*_*n*_(α) is the alpha-band power and *P*_*n*_(*t*), which equals {*P*_*n*_(θ) + *P*_*n*_(α) + *P*_*n*_(β)}, is the total power at electrode *n*. Instead of calculating the reciprocal of alpha-band power, 1/*P*_*n*_(α), based on a negative correlation between cortical activity and alpha-band power (Coan and Allen, 2004; Oakes et al., 2004; Pizzagalli et al., 2005; Koslov et al., 2011), we calculated the reciprocal of the alpha-band power normalized to the total power of each participant, 1/{*P*_*n*_(α)/*P*_*n*_(*t*)}, to reduce inter-individual variation arising from differences in skull thickness (Eshel et al., 1995; Allen et al., 2004; Hagemann et al., 2008). We excluded mean power in the delta band (*P*_*n*_(δ), 0.5–4 Hz) when calculating total power of the *P*_*n*_(*t*), because the experiment was carried out during daytime when participants do not sleep. In real life, the delta wave of EEG can be observed only in the deep-sleep state. Thus, the delta band of EEG measured during daytime can be regarded as noise and filtered. Using this method, *P*_*n*_(*r*) is proportional to the cortical activity around electrode number *n.*

**FIGURE 3:**
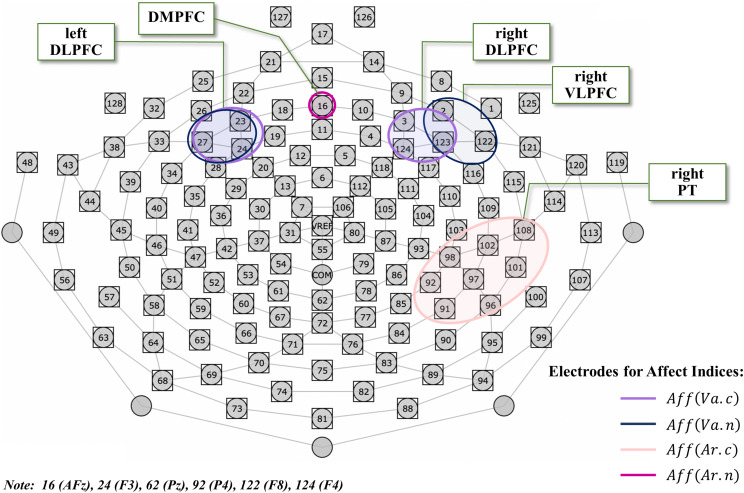
The HydroCel GSN 128-channel map used in this experiment. The electrodes used for EEG analysis are surrounded by colored lines corresponding to each of the specific affect indices, where light violet represents the conventional valence index comprising left DLPFC (23-24-27) and right DLPFC (3-123-124); dark blue represents the novel valence index comprising left DLPFC (23-24-27) and right VLPFC (2-122-123); light pink represents the conventional arousal index comprising right PT (91-92-96-97-98-101-102-108); and purple represents the novel arousal index comprising DMPFC (16). The electrode on vertex Cz (REF) was used as reference, and the electrode at COM between Cz and Pz as the ground. DLPFC, dorsolateral prefrontal cortex; VLPFC, ventrolateral prefrontal cortex; PT, parietotemporal; DMPFC, dorsomedial prefrontal cortex.

To investigate affect, we defined a conventional index of affect grounding on the limbic system including amygdala, which has been the focus of majority of studies (Ahern and Schwartz, 1985; Heller et al., 1997; Phelps and Anderson, 1997; Davidson and Irwin, 1999). Specifically, we defined the conventional index for affective valence as *Aff(Va.c)*, based on differential lateralization for positive and negative emotions (Ahern and Schwartz, 1985; Heller et al., 1997). This was calculated by subtracting the natural-log-transformed *P*_*n*_(*r*) on the scalp near the right DLPFC from that near the left DLPFC. We clustered three electrodes (23-24-27) including F3 on the scalp near the left DLPFC, and three electrodes (3-123-124) including F4 on the scalp near the right DLPFC, according to the reference system of the HydroCel GSN 128-channel map and the international 10-10 system (see Figure 3 and Equation (2)). We also defined a novel index of affect, focusing on the role of the prefrontal cortex (PFC) regions in emotion processing (Anderson et al., 2003). We defined the novel index for affective valence as *Aff(Va.n)*, based on a fMRI study (Dolcos et al., 2004), which was calculated by subtracting the natural-log-transformed *P*_*n*_(*r*) on the scalp near the right VLPFC from that near the left DLPFC. We clustered three electrodes (2-122-123) including F8 on the scalp near the right VLPFC. The clustering of the scalp near the left DLPFC was the same as that of the conventional index (see Figure 3 and Equation (3)). A more positive value of *Aff(Va.c)* or *Aff(Va.n)* reflects a positive affective valence state (in other words, a more pleasant state), whereas a more negative value represents the opposite. To examine affective arousal, we defined a conventional index as *Aff(Ar.c)*, which was calculated from the *P*_*n*_(*r*) in the region of the right PT since the PT is responsible for affective arousal (Heller et al., 1997). We clustered eight electrodes (91-92-96-97-98-101-102-108) including P4 and T8 for right PT (see Figure 3 and Equation (4)). We also defined a novel index for affective arousal as *Aff(Ar.n)*, which was calculated from the *P*_*n*_(*r*) on electrode number 16 near the DMPFC based on previous fMRI studies (Dolcos et al., 2004; Grimm et al., 2006) (see Section “Introduction,” Figure 3, and Equation (5)). Thus, a greater value of *Aff(Ar.c)* or *Aff(Ar.n)* represents a higher affective arousal state. As shown in Figure 3, we measured and analyzed the EEG for frontal and right parietotemporal regions together.

(1) $$P_{n}\,\left(r\right)\equiv \frac{1}{P_{n}\,\left(\alpha \right)/\left\{P_{n}\,\left(\theta \right)+P_{n}\,\left(\alpha \right)+P_{n}\,\left(\beta \right)\right\}}\equiv \frac{1}{P_{n}\,\left(\alpha \right)/p_{n}\,\left(t\right)}=\frac{p_{n}\,\left(t\right)}{p_{n}\,\left(\alpha \right)}$$

(2) $$Aff\left(Va.c\right)\equiv [ln^{\left(P_{23}\,\left(r\right)\right)}-ln^{\left(P_{3}\,\left(r\right)\right)}+ln^{\left(P_{24}\,\left(r\right)\right)}-ln^{\left(P_{124}\,\left(r\right)\right)}+ln^{\left(P_{27}\,\left(r\right)\right)}-ln^{\left(P_{123}\,\left(r\right)\right)}]/3$$

(3) $$Aff\left(Va.n\right)\equiv \left[ln^{(P_{23}}\left(r\right)\right)-ln^{\left(P_{2}\,\left(r\right)\right)}+ln^{\left(P_{24}\,\left(r\right)\right)}-ln^{\left(P_{123}\,\left(r\right)\right)}+ln^{\left(P_{27}\,\left(r\right)\right)}-ln^{\left(P_{122}\,\left(r\right)\right)}]/3=\frac{\left[l\,n\,\left\{\frac{P_{23}\,\left(r\right)}{P_{2}\,\left(r\right)}\right\}+l\,n\,\left\{\frac{P_{24}\,\left(r\right)}{P_{123}\,\left(r\right)}\right\}+l\,n\,\left\{\frac{P_{27}\,\left(r\right)}{P_{122}\,\left(r\right)}\right\}\right]}{3}$$

(4) $$Aff\left(Ar.c\right)\equiv \frac{\left\{\begin{array}{c} P_{91}\,\left(r\right)+P_{92}\,\left(r\right)+P_{96}\,\left(r\right)+P_{97}\,\left(r\right)\\ +P_{98}\,\left(r\right)+P_{101}\,\left(r\right)+P_{102}\,\left(r\right)+P_{108}\,\left(r\right) \end{array}\right\}}{8}$$

(5) $$Aff\left(Ar.n\right)\equiv P_{16}\left(r\right)$$

#### Stroop Task Analyses

The *RTs* for correct responses were labeled as *RT*_*correct*, while those for incorrect responses were labeled as *RT*_*incorrect*. Similarly, the *RTs* for congruent stimuli were labeled as *RT*_*cong*, while those for incongruent stimuli were labeled as *RT*_*incong*. *ErR* was defined as the number of incorrect responses for the congruent (incongruent) stimuli divided by 320 and labeled as *ErR*_*cong*(*ErR*_*incong*). Trials with no response were excluded when calculating the *RT*. However, such trials were also considered incorrect when computing the *ErR*. The six indices defined above were calculated for each participant by taking the averages of their corresponding responses to the 640 stimuli.

### Statistical Analysis

Based on an experimental design using repeated-measures analysis of variance (ANOVA) for within-between interaction with a medium effect size (Cohen’s *d* of 0.5), using G^∗^Power 3.1.9.2 (Faul et al., 2007), we calculated a minimum sample size of 17 per group to achieve 80% power (*α* = 0.05). This sample size is comparable with that of previous similar experimental studies (e.g., Inzlicht and Gutsell, 2007). Even though the within-subject design requires more small minimum sample sizes compared with the between-subject design, we employed the between-subject design because the performance of color-word Stroop task depends on the order, i.e., the performance of second trial will be better than the first under same cognitive capacity conditions.

As the indices for both groups that evaluated affect in Task 1 and cognitive performance in task 2 were normally distributed according to the Kolmogorov–Smirnov tests (all *p* > .05), we employed a 2 × 2 (session × group) repeated-measures ANOVA to test our hypotheses while excluding the influence of different baseline levels for each participant in task 1. We explored the interaction effects between sessions (baseline vs. ArtView) and groups (AbNP vs. AbEX) on affective valence and affective arousal in task 1. This approach is used to test whether there was a difference in affect between the baseline and ArtView sessions as a function of viewing the two kinds of abstract art. In Task 2, we investigated the main effects of the responses (correct vs. incorrect), stimuli (congruent vs. incongruent), and groups (AbNP vs. AbEX) on *RT*and *ErR* to test the impact of the differential affect induced in the two groups during task 1 on *RT* and *ErR* when performing the Stroop task. In addition, correlations between the two performance indices, *RT* and *ErR*, in task 2 were analyzed. Effect sizes were estimated as partial eta-squared (*η_*p*_^2^*). Levels of significance and marginal significance were set at *α* = 0.05 and *α* = 0.07, respectively. SPSS 24 (IBM, Armonk, NY, United States) was used to perform the statistical analyses.

## Results

### Task 1: Influence of Visual Art on Affect

**FIGURE 4:**
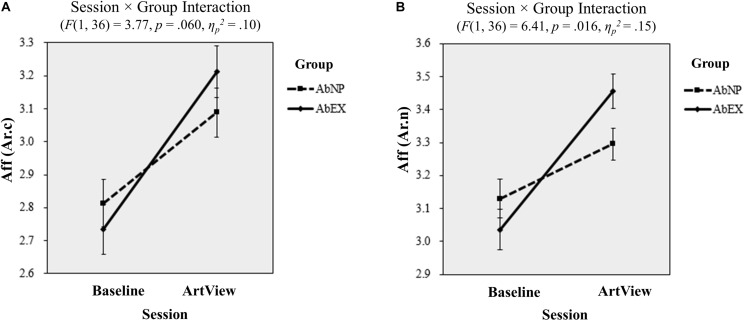
Results of the EEG analyses from task 1 for affective arousal: the effect of viewing abstract artwork (vs. baseline) on **(A)** *Aff*(*Ar*.*c*) and **(B)** *Aff*(*Ar*.*n*) is shown for the AbNP (dashed line) and AbEX groups (solid line), respectively. Error bars show standard errors of the mean. The terms *Aff*(*Ar*.*c*) and *Aff*(*Ar*.*n*) represent the conventional and novel indices for arousal respectively used in the EEG analyses. ArtView, artwork-viewing session; AbNP group, abstract neoplastic artwork-viewing group; AbEX group, abstract expressionist artwork-viewing group.

A 2 (baseline vs. ArtView) × 2 (AbNP vs. AbEX) repeated-measures ANOVA showed no interaction for the conventional affective valence index *Aff(Va.c)* (*F*(1, 36) = 1.11, *p* = 0.300, *η_*p*_*^2^ = 0.03), or for the novel affective valence index *Aff(Va.n)* (*F*(1, 36) = 1.38, *p* = 0.248, *η_*p*_*^2^ = 0.04). However, for conventional affective arousal index *Aff(Ar.c)*, the session × group interaction was marginally significant (*F*(1, 36) = 3.77, *p* = 0.060, *η_*p*_*^2^ = 0.10). This indicated that the increase in affective arousal from the baseline to the ArtView session was larger for the AbEX group (*M*_baseline_ = 2.73, 95% CI = [2.57, 2.88] vs. *M*_ArtView_ = 3.19, 95% CI = [3.03, 3.35]) when compared with the AbNP group (*M*_baseline_ = 2.83, 95% CI = [2.68, 2.98] vs. *M*_ArtView_ = 3.09, 95% CI = [2.94, 3.24]) (see Figure 4A). For *Aff(Ar.n)*, the interaction was also significant (*F*(1, 36) = 6.41, *p* = 0.016, *η_*p*_*^2^ = 0.15), indicating that the same pattern held true for the novel affective arousal index (AbEX: *M*_baseline_ = 3.05, 95% CI = [2.92, 3.17] vs. *M*_ArtView_ = 3.45, 95% CI = [3.35, 3.56]; AbNP: *M*_baseline_ = 3.13, 95% CI = [3.02, 3.25] vs. *M*_ArtView_ = 3.30, 95% CI = [3.20, 3.39]) (see Figure 4B). This means that the affective arousal was induced to a higher degree in participants who viewed abstract art of expressionism as opposed to those who viewed neoplasticism.

To inspect the influence of the two opposing styles of abstract art (i.e., expression vs. suppression) on the viewers’ affect, we analyzed the EEG power in time-frequency domain, i.e., short-time Fourier transform (STFT) of EEG, by using EEGLAB and MATLAB. We plotted the individual spectrograms belonging to four sites (left DLPFC and right DLPFC for affective valence and right PT and DMPFC for affective arousal) for baseline and ArtView sessions and for both AbNP and AbEX groups. The number of individual spectrograms was 304 (i.e., 38 participants × 2 sessions [baseline and ArtView] × 4 sites [left DLPFC, right DLPFC, right PT, and DMPFC]). These spectrograms are available in the following URL^2^.

To enhance the discrimination of alpha power reduction, i.e., to increase the frequency resolution of the spectrogram, the FFT was applied to 4-s windows with a 2-s overlap, instead of 1-s window with a 0.5-s overlap as described in Section “EEG analysis.” Since the present study does not intend to examine event-related potential (ERP), where the ensemble average will improve the signal-to-noise ratio as the number of trials increase, we chose one segment each corresponding to the baseline and ArtView sessions of the two groups, instead of 10 segments from the baseline session and 20 from the ArtView session (see Section “EEG analysis”). The reason is that the spectrogram of the spontaneous EEG in the present study will tend to be white noise as the number of segments increase, because the EEG in these segments are not synchronized. Moreover, to ensure the fairness of EEG power comparison, the time range of each segment was set to be the same in each spectrogram. In this study, we restricted the time range of the 304 spectrograms to 10–40 s because some of the individual spectrograms had severe noise when the range exceeded 40 s.

**FIGURE 5:**
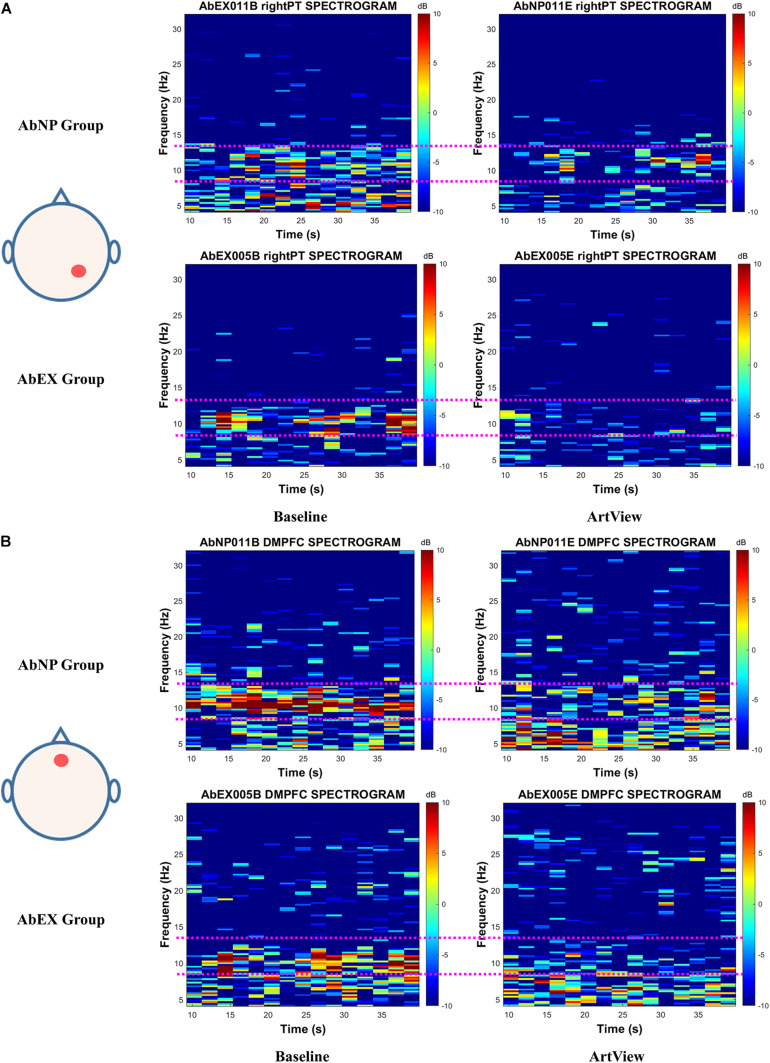
The EEG spectrogram for a typical participant of the AbNP group and that of the AbEX group for **(A)** *Aff(Ar.c)* represents the effect of viewing abstract artwork (vs. baseline) on right parietotemporal cortex (PT). For **(B)** *Aff(Ar.n)* represents the effect of viewing abstract artwork (vs. baseline) on the dorsomedial prefrontal cortex (DMPFC). The alpha power suppression in ArtView (vs. baseline) session is visually obvious, especially in AbEX group for *Aff(Ar.c)* and for *Aff(Ar.n)*. This result is consistent with the results of statistical analysis in Figure 4A for *Aff(Ar.c)* and in Figure 4B for *Aff(Ar.n)*, i.e., the session × group interaction was significant for novel as well as conventional indices of affective arousal. The terms *Aff*(*Ar*.*c*) and *Aff*(*Ar*.*n*) represent the conventional and the novel indices for arousal, respectively. ArtView, AbNP group, and AbEX group have the same meanings described in the legend of Figure 4. The pink dotted line in each spectrogram represents the boundary of alpha band EEG, i.e., 8–13 Hz. The color bar represents the strength of EEG power in dB. Each spectrogram represents EEG power, which depends on time and frequency.

As an illustrative example, a typical participant was selected for each group. At first, three participants for each group were selected randomly. After then, the participant whose difference of *Aff(Ar.n)* between baseline and ArtView sessions was the largest among the three was chosen from each group, because the effect of abstract artwork on this index was significant as described in Figure 4. As a result, the 11th participant was selected for AbNP group and the 5th participant for AbEX group. To evaluate the effect of the artwork on the conventional index of affective arousal, i.e., *Aff(Ar.c)*, we analyzed the spectrogram for the typical participant from the AbNP and AbEX groups and represented the results in Figure 5A for right PT. We also evaluated for the novel index, i.e., *Aff(Ar.n)*, and represented the spectrogram in Figure 5B for DMPFC. Each title of the individual spectrogram comprises the participant ID, the session, and the site corresponding to when and where the spectrogram was evaluated. For example, the title “AbNP011B right PT SPECTROGRAM” in Figure 5A represents that the spectrogram was evaluated at the site of the right PT during the baseline session for the 11th participant of the AbNP group. The title “AbEX005E DMPFC SPECTROGRAM” represents that the spectrogram evaluated at DMPFC during the experiment, i.e., ArtView, session for the 5th participant of the AbEX group. The pink dotted line in each spectrogram represents the boundary of alpha band EEG, which is 8–13 Hz. The color bar represents the strength of EEG in decibels (dB). Each spectrogram represents EEG power (color in dB) depending on time (10–40 s) and frequency (4–32 Hz). Results show that the alpha power suppression in ArtView session is evident when compared with the baseline session, especially in the AbEX group for *Aff(Ar.c)* as shown in Figure 5A and for *Aff(Ar.n)* as shown in Figure 5B. These results also support the results of the statistical analysis, which was based on the alpha power, as described in Figure 4A for *Aff(Ar.c)* and in Figure 4B for *Aff(Ar.n)*, i.e., the session × group interaction was significant for novel as well as conventional indices of affective arousal.

**FIGURE 6:**
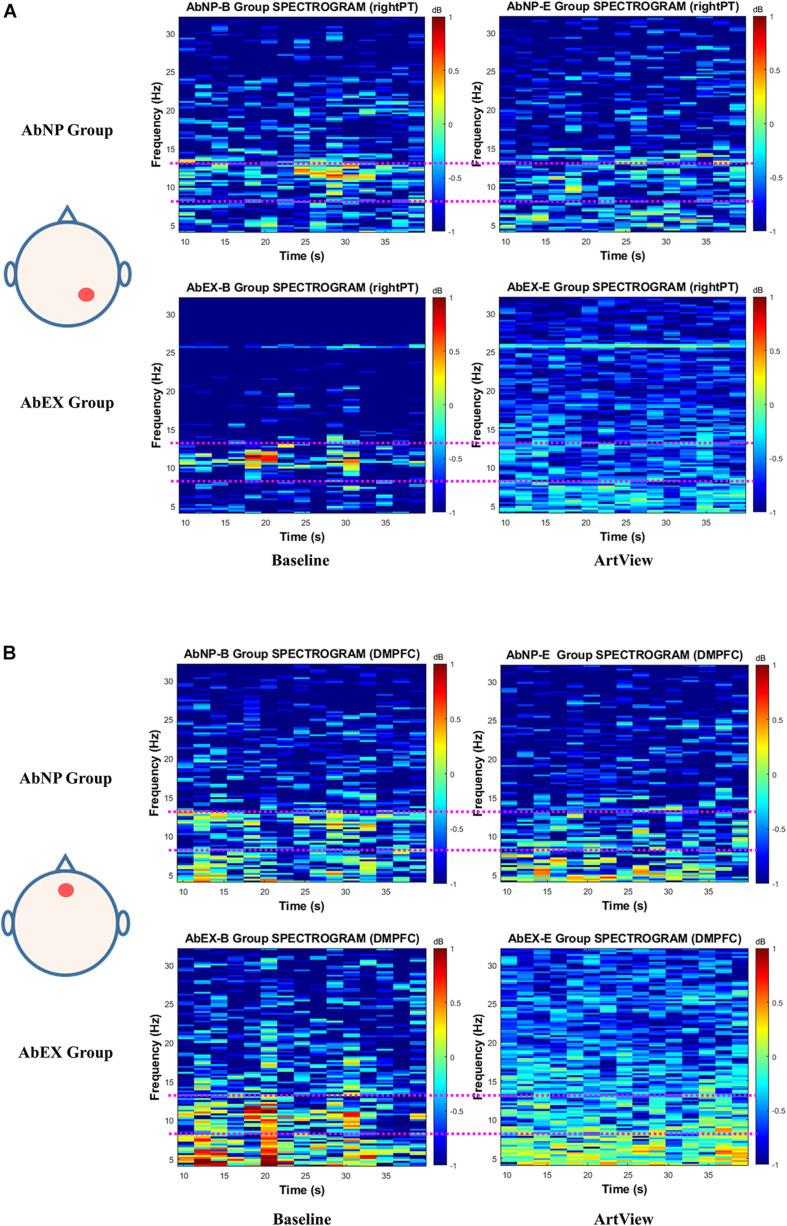
The EEG spectrogram for a mean of the AbNP group and that of the AbEX group, depending on baseline and ArtView sessions, for **(A)** *Aff(Ar.c)* represents the effect of viewing abstract artwork (vs. baseline) on right parietotemporal cortex (PT). For **(B)**, *Aff(Ar.n)* represents the effect of viewing abstract artwork (vs. baseline) on the dorsomedial prefrontal cortex (DMPFC). The trend of the difference of alpha power reduction in group spectrograms is similar to that of the typical spectrograms in Figure 5. This result is consistent with the results of statistical analysis in Figure 4A for *Aff(Ar.c)* and in Figure 4B for *Aff(Ar.n)*. The terms*Aff*(*Ar*.*c*), *Aff*(*Ar*.*n*), ArtView, AbNP group, and AbEX group, the pink dotted line, and the color bar in each spectrogram have the same meanings described in the legend of Figure 5.

Next, we calculated the mean spectrogram for baseline and ArtView sessions for each group. We defined these as “group spectrograms” as described in Figure 6 for affective arousal. Even though the difference of alpha power reduction in group spectrograms of Figure 6 was not as apparent as in typical spectrograms shown in Figure 5, the trend was similar.

### Task 2: Performance in the Stroop Task

#### Reaction Time

**FIGURE 7:**
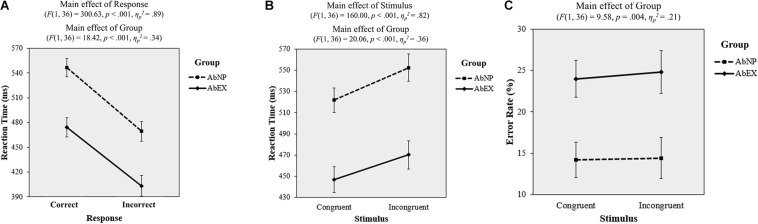
Plots of the results of task 2 for both the AbNP and AbEX groups, including reaction time, by **(A)** response type or **(B)** stimulus type, and **(C)** error rate by stimulus type. Error bars indicate standard errors of the mean. AbNP group and AbEX group have the same meanings described in the legend of Figure 4.

A 2 (correct vs. incorrect) × 2 (AbNP vs. AbEX) repeated-measures ANOVA was conducted to analyze *RT*. The main effect of response was significant (*F*(1, 36) = 300.63, *p* < 0.001, *η_*p*_*^2^ = 0.89), with *RT*_*incorrect* (*M* = 436.38 ms, 95% CI = [418.71, 454.04]) being faster than *RT*_*correct* (*M* = 510.49 ms, 95% CI = [494.38, 526.60]). This means that when participants make quick decisions, they run the risk of committing mistakes regardless of how much cognitive resources they have remaining. The main effect of group was also significant (*F*(1, 36) = 18.42, *p* < 0.001, *η_*p*_*^2^ = 0.34), with the *RT* of the AbEX group (*M* = 438.86 ms, 95% CI = [415.14, 462.57]) being faster than that of the AbNP group (*M* = 508.01 ms, 95% CI = [485.52, 530.51]). This means that if participants’ cognitive resources are depleted, they are more likely to make quick decisions regardless of their mistake. Consistent with these two results, the response × group interaction was not significant (*F*(1, 36) = 0.53, *p* = 0.472, *η_*p*_*^2^ = 0.01) (see Figure 7A).

A 2 (congruent vs. incongruent) × 2 (AbNP vs. AbEX) repeated-measures ANOVA was conducted on *RT*. The main effect of the stimulus (*F*(1, 36) = 160.00, *p* < 0.001, *η_*p*_*^2^ = 0.82) was significant, with *RT*_*incong* (*M* = 511.35 ms, 95% CI = [492.55, 530.15]) being slower than *RT*_*cong* (*M* = 484.36 ms, 95% CI = [467.31, 501.41]). This means that as the cognitive task become difficult, participants tend to take longer to make decisions regardless of the amount of their cognitive resources remaining. The main effect of group was also significant (*F*(1, 36) = 20.06, *p* < 0.001, *η_*p*_*^2^ = 0.36), with the *RT* of the AbEX group (*M* = 458.51 ms, 95% CI = [432.65, 484.36]) being faster than that of the AbNP group (*M* = 537.20 ms, 95% CI = [512.68, 561.73]). This means that if participants’ cognitive resources are depleted, they are more likely to make quicker decisions regardless of the difficulty of cognitive task. Consistent with these two results, the stimulus × group interaction was not significant (*F*(1, 36) = 2.98, *p* = 0.093, *η_*p*_*^2^ = 0.08) (see Figure 7B).

#### Error Rate

A 2 (congruent vs. incongruent) × 2 (AbNP vs. AbEX) repeated-measures ANOVA was conducted on *ErR*. The result indicated a significant main effect (*F*(1, 36) = 9.58, *p* = 0.004, *η_*p*_*^2^ = 0.21), with the effect of *ErR* on the AbEX group (*M* = 24.40%, 95% CI = [19.60, 29.20]) being higher than that of the AbNP group (*M* = 14.31%, 95% CI = [9.75, 18.86]). However, neither the main effect of the stimulus type (*F*(1, 36) = 0.48, *p* = 0.495, *η_*p*_*^2^ = 0.01) nor the interaction were significant (*F*(1, 36) = 0.15, *p* = 0.705, *η_*p*_*^2^ < 0.01) (see Figure 7C). This means that if participants deplete their cognitive resources, then they tend to make mistakes regardless of the difficulty of cognitive task.

#### Correlations Between *RT*and *ErR*

There were significant negative correlations between *RT* and *ErR* in both the AbNP group (congruent: *r* = −0.46, *p* = 0.041; incongruent: *r* = −0.47, *p* = 0.037) and the AbEX group (congruent: *r* = −0.88, *p* < 0.001; incongruent: *r* = −0.90, *p* < 0.001). This means that if participants make quick decisions, they are more likely to commit mistake. This trend is more evident as their cognitive resources deplete more.

### Subjective Ratings for Abstract Artwork

A one-way ANOVA was conducted on the subjective ratings provided by participants from the manipulation check groups. Participants who viewed AbEX rated the four items, i.e., “affective arousal,” “dynamicity,” “complexity,” and “emotional infusion” significantly higher than those who viewed AbNP. However, there was no significant difference between the two groups for affective valence. Table 1 details the means, 95% confidence intervals, and statistical tests for these comparisons.

**TABLE 1:** Means and confidence intervals for the subjective ratings of the abstract artwork.

	AbNP group (*n* = 33)		AbEX group (*n* = 33)		Comparison	
	*M*	95% CI	*M*	95% CI	*F*(1, 64)	*p*
Arousal	3.60	[3.00, 4.20]	4.72	[4.24, 5.20]	8.91	0.004
Dynamicity	3.27	[2.79, 3.75]	5.38	[4.94, 5.81]	43.81	<0.001
Complexity	3.74	[3.25, 4.23]	6.85	[6.51, 7.18]	114.08	<0.001
Emotional infusion	3.88	[3.21, 4.54]	5.19	[4.63, 5.76]	9.43	0.003
Valence	3.83	[3.21, 4.45]	3.85	[3.42, 4.28]	<1.00	0.961

**AbNP*, artwork of Mondrian in the style of neoplasticism; *AbEX*, artwork of Kandinsky in the style of expressionism; *CI*, confidence interval; *M*, mean.*

## Discussion

The primary aim of this study was to examine whether changes in consumer’s affect induced by opposing styles of visual art (expressing vs. suppressing) on product packaging differ, if so, to investigate how the differential changes in consumer’s affect induced by the two styles of visual art influence the way information is processed in purchase decision-making.

Proving our first hypothesis, the results showed that observing AbEX increased affective arousal when compared with observing AbNP. This finding was apparent in both the conventional and novel affective arousal indices, i.e., *Aff(Ar.c)* and *Aff(Ar.n)* (see these definitions in Section “EEG analysis”), based on EEG analyses of task 1, which included baseline and ArtView sessions. This implies that affective arousal is induced more in consumers who view abstract art of expressionism as opposed to neoplasticism.

Our results are in line with previous findings. Both the right PT cortex (Heller et al., 1997) and DMPFC (Dolcos et al., 2004; Grimm et al., 2006) were involved in the induction of affective arousal. We hypothesized that abstract artwork expressing the emotion and gestural movement of the artist, represented by dynamic elements in the artwork of Kandinsky, would evoke higher affective arousal. Our EEG data, as well as the subjective ratings of the participants, provide empirical evidence to support this hypothesis. Our results are also consistent with studies showing that the abstract artwork by Kandinsky ranks high in both complexity (Wohlwill, 1968) and affective arousal (Hagtvedt et al., 2008). Furthermore, it has been demonstrated that viewing pictures which induced affective arousal increased motor cortex excitability (Hajcak et al., 2007), and that dynamic elements in the abstract artwork by Franz Kline activated the motor cortex (Sbriscia-Fioretti et al., 2013). Thus, higher levels of dynamic properties in Kandinsky’s artwork compared with Mondrian’s artwork might have facilitated embodied simulation of the gestural movements of the artist (Gallese and Sinigaglia, 2011). The results of this study showed an elevated affective arousal state of the AbEX group compared with that of the AbNP group and can be explained by the embodied simulation theory, i.e., Kandinsky’s emotion was simulated within the somatosensory systems of the AbEX group. This means that affective arousal will be induced more in consumers who view visual art that is expressing as opposed to suppressing its artist’s affect.

Consistent with our second hypothesis, we found that participants who viewed artwork by Kandinsky but not Mondrian performed poorly in the subsequent Stroop task. We confirmed that affective arousal as well as affective valence may play a grand claim in determining the impact of affect on cognition (Mitchell and Phillips, 2007). The higher affective arousal induced by AbEX compared with AbNP might deplete the necessary cognitive resources needed for performing the Stroop task (Pallak et al., 1975). We infer that the lower performance associated with viewing AbEX than AbNP is due to the unavoidable use of a heuristic, rather than a systematic, strategy for decision-making due to a deficiency in cognitive resources caused by the enhanced affective arousal (Paulhus and Lim, 1994; Pham, 1996; Fedorikhin and Patrick, 2010). Significant evidence exists to support this conjecture. First, the reaction time (*RT*) indices in our data (see Figure 7A) indicate that the AbEX group responded faster than the AbNP group regardless of response type (correct vs. incorrect), and the *RT* for the incorrect answers is shorter than that of the correct answers regardless of group type (AbNP vs. AbEX). This means that if consumers’ cognitive resources are depleted, they are likely to make quick decisions regardless of their mistake and while making quick decisions, they tend to commit mistakes regardless of the amount of their cognitive resources remaining. Second, the *RT* indices in our data (see Figure 7B) indicate that the AbEX group responded faster than the AbNP group regardless of stimulus type (congruent vs. incongruent) and the *RT* for the incongruent stimuli is longer than that of the congruent stimuli regardless of group type (AbNP vs. AbEX). This implies that if consumers’ cognitive resources are depleted, they are likely to make quick decisions regardless of the difficulty of cognitive task and as the cognitive task becomes more difficult, they tend to take longer time to make decisions regardless of the amount of their cognitive resources remaining. Third, the error rate (*ErR*) was found to be higher in the AbEX group than in the AbNP group for both congruent and incongruent stimuli (see Figure 7C). This suggests that if consumers deplete their cognitive resources, then they tend to make mistakes regardless of the difficulty of cognitive task. It is important to note that faster *RT* may be the cause of higher *ErR*, a phenomenon that is typical of heuristic decision-making. This result is consistent with the negative correlations between *RT* and *ErR* in both the AbNP and AbEX groups. This supports previous studies where it is seen that the available time is analogous to cognitive capacity or the ability to process information (Suri and Monroe, 2003).

The question then becomes how and why affective arousal reduces the consumers’ cognitive capacity and leads to heuristic processing. Earlier research suggests various reasons for the depletion of cognitive resources (Fedorikhin and Patrick, 2010). Consistent with the results of this study (i.e., higher affective arousal group, AbEX, performed poorly in the subsequent Stroop task), individuals in a high-arousal state are more likely to be persuaded by peripheral cues than those in a low-arousal state (Sanbonmatsu and Kardes, 1988). In addition, they are more likely to suppress reliance on cues that are cognitive-capacity demanding (Pham, 1996; Fedorikhin and Patrick, 2010). According to the dynamic complexity model, highly aroused individuals also tend to reduce the cognitive complexity of judgments by focusing on the primary dimension and discarding the secondary dimension (Paulhus and Lim, 1994).

This research has a potential limitation. Only male participants with relatively short hair were recruited to reduce the time required to adjust the 128 electrodes and to maintain homogeneity. For future research, the study can include female participants. To evaluate cognitive task performance, other measures could also be considered such as n-back task, digit span task, digit symbol test, and word fluency test, which have been widely used as psychometric tests in cognitive psychology studies (Gajewski et al., 2018).

In summary, the current study proves that viewing abstract expressionist artwork (by Kandinsky) induces a far stronger affect in the viewer than viewing neoplastic artwork (by Mondrian), particularly in terms of affective arousal. This results in a downstream effect that diminishes performance in subsequent cognitive tasks, as demonstrated by the Stroop task. One possible reason for worse performance is the depletion of cognitive resources. Such depletion occurs as a result of a heightened affective arousal caused by the greater complexity and dynamicity of abstract artwork that attempt to express, rather than suppress, the emotions of the artist. This paper is the first known attempt to demonstrate how the changes in consumer’s affect induced by visual art with different styles designed on product or product packaging influence their decision-making under time constraint (e.g., while purchasing). Our findings indicate that if an art-based design on product or product packaging expresses (vs. suppress) the artist’s emotion (e.g., includes dynamic components), then the design may deplete consumer’s cognitive resources, thereby leading to heuristic decision-making and resulting in inadvertent mistakes while purchasing. This research provides a foundation to build a solid theory on the relationship between abstract art inducing high affective arousal and leading to heuristic decision-making. The findings are especially relevant in the field of neuromarketing when the abstract art is used in the packaging of products (Breiter et al., 2015; Ramsøy et al., 2018).

## Data Availability Statement

The datasets presented in this study can be found in online repositories. The names of the repository/repositories and accession number(s) can be found in the article/Supplementary Material.

## Ethics Statement

The studies involving human participants were reviewed and approved by Korea Research Institute of Standards and Science. The patients/participants provided their written informed consent to participate in this study. Written informed consent was obtained from the individual(s) for the publication of any potentially identifiable images or data included in this article.

## Author Contributions

YRK and YEK designed the study under the supervision of KP and conducted the experiments under the supervision of W-SK. YRK and WY drafted the manuscript under the supervision of KP and W-SK. KP supervised statistical analyses, interpreted the data, and provided critical revisions with respect to research in marketing. DH analyzed the EEG data under the supervision of W-SK. W-SK supervised the measurement and analysis of the EEG and provided critical revisions regarding research in neuroscience. All the authors involved approved the final manuscript prior to submission.

## Conflict of Interest

The authors declare that the research was conducted in the absence of any commercial or financial relationships that could be construed as a potential conflict of interest.

## Footnotes

1 http://www.moma.org/collection/artists/2981

2 https://drive.google.com/open?id=1ag4CUi9vOHT39q95ob7XCAQbhWP51vkI
